# Virtual Risk Management—Exploring Effects of Childhood Risk Experiences through Innovative Methods (ViRMa) for Primary School Children in Norway: Study Protocol for the ViRMa Project

**DOI:** 10.2196/45857

**Published:** 2023-06-07

**Authors:** Ellen Beate Hansen Sandseter, Ole Johan Sando, Håvard Lorås, Rasmus Kleppe, Lise Storli, Mariana Brussoni, Anita Bundy, David C Schwebel, David J Ball, Monika Haga, Helen Little

**Affiliations:** 1 Department of Physical Education and Health Queen Maud University College of Early Childhood Education Trondheim Norway; 2 Department of Teacher Education Norwegian University of Science and Technology Trondheim Norway; 3 Department of Pediatrics, Human Early Learning Partnership School of Population and Public Health University of British Columbia Vancouver, BC Canada; 4 British Columbia Children’s Hospital Research Institute Vancouver, BC Canada; 5 Department of Occupational Therapy Colorado State University Fort Collins, CO United States; 6 Department of Psychology University of Alabama at Birmingham Birmingham, AL United States; 7 Department of Science and Technology, Centre for Decision Analysis and Risk Management Middlesex University London United Kingdom; 8 School of Education Macquarie University Sydney Australia

**Keywords:** anxiety, childhood education, childhood risk experiences, children, development, eye tracking, injury prevention, injury, motion capturing, physical activity, prevention, questionnaire, resilience, risk management, risky play, social skills, validation, virtual reality, VR, well-being, willingness, Xsens

## Abstract

**Background:**

Research indicates that risky play benefits children’s risk assessment and risk management skills and offers several positive health effects such as resilience, social skills, physical activity, well-being, and involvement. There are also indications that the lack of risky play and autonomy increases the likelihood of anxiety. Despite its well-documented importance, and the willingness of children to engage in risky play, this type of play is increasingly restricted. Assessing long-term effects of risky play has been problematic because of ethical issues with conducting studies designed to allow or encourage children to take physical risks with the potential of injury.

**Objective:**

The Virtual Risk Management project aims to examine children’s development of risk management skills through risky play. To accomplish this, the project aims to use and validate newly developed and ethically appropriate data collection tools such as virtual reality, eye tracking, and motion capturing, and to provide insight into how children assess and handle risk situations and how children’s past risky play experiences are associated with their risk management.

**Methods:**

We will recruit 500 children aged 7-10 years and their parents from primary schools in Norway. Children’s risk management will be measured through data concerning their risk assessment, risk willingness, and risk handling when completing a number of tasks in 3 categories of virtual reality scenarios: street crossing, river crossing, and playing on playground equipment. The children will move around physically in a large space while conducting the tasks and wear 17 motion-capturing sensors that will measure their movements to analyze motor skills. We will also collect data on children’s perceived motor competence and their sensation-seeking personality. To obtain data on children’s risk experiences, parents will complete questionnaires on their parental style and risk tolerance, as well as information about the child’s practical risk experience.

**Results:**

Four schools have been recruited to participate in data collection. The recruitment of children and parents for this study started in December 2022, and as of April 2023, a total of 433 parents have consented for their children to participate.

**Conclusions:**

The Virtual Risk Management project will increase our understanding of how children’s characteristics, upbringing, and previous experiences influence their learning and ability to handle challenges. Through development and use of cutting-edge technology and previously developed measures to describe aspects of the children’s past experiences, this project addresses crucial topics related to children’s health and development. Such knowledge may guide pedagogical questions and the development of educational, injury prevention, and other health-related interventions, and reveal essential areas for focus in future studies. It may also impact how risk is addressed in crucial societal institutions such as the family, early childhood education, and schools.

**International Registered Report Identifier (IRRID):**

DERR1-10.2196/45857

## Introduction

### Background

How children learn to handle risk and avoid injuries is poorly understood by researchers. Injuries remain the leading cause of child death and a leading cause of hospitalization in high-income nations despite widespread prevention efforts. Among the leading mechanisms of injury are motor vehicle crashes (being a passenger in a car) [1] and falls (eg, from high playground equipment) [2]. Drowning, pedestrian, and cycling injuries also represent threats of serious injury and death [3]. Improved car safety has resulted in significant improvements in injury rates, but injury prevention efforts must be multifaceted, addressing the physical environment and individual risk factors [4]. One novel and promising approach to child injury prevention may be through improving children’s own ability to assess risk. In this regard, *play* appears to be an ideal context for children’s learning, as the pretend or nonliteral aspect allows the players to test out risky behavior, situations, or actions without the severe consequences of real life. *Risky play* has therefore emerged as a topic of interest for researchers, parents, teachers, policy makers, authorities, and others working with children over the last 20 years. A growing body of research investigates various aspects of risk-taking, including playful activities, and indicates that the concept reflects a basic aspect of human life.

The link between childhood risky play experiences and subsequent ability to cope with hazards of everyday life warrants investigation. A common definition of risky play is “thrilling and exciting forms of physical play that involve uncertainty and a risk of physical injury” [5]. Through observations and interviews with children and early childhood education and care (ECEC) staff, Kleppe et al [6] and Sandseter et al [7,8] identified eight categories of risky play: (1) play with great heights, (2) play with high speed, (3) play with dangerous tools, (4) play near dangerous elements, (5) rough-and-tumble play, (6) play where children go exploring alone, (7) play with impact, and (8) vicarious risk.

Qualitative studies suggest that children develop skills and learn to cope with risk through risky play that translates to real life and is retained for a lifetime [9-14]. Engagement in risk-taking activities leads to brain development, for example, and enhances the ability of 18- to 19-year-olds to predict outcomes, supporting the hypothesis that children and youth will learn risk assessment through risk experiences [15,16]. Moreover, there are indications that risky play in early childhood has a positive impact on the development of children’s own risk management skills [11,17]. Researchers also suggest that the ability to handle risk is a favorable evolutionary trait [18] and that outdoor risky play offers several positive health effects for children such as resilience, social skills, physical activity, well-being, and involvement [19,20]. In addition, there are indications that the lack of risky play and autonomy increases the likelihood of anxiety, both in childhood [21-24] and subsequently in adolescence and adulthood [18,25-29].

Children’s affective, cognitive, perceptual, and motor systems are all involved in children’s behavior and activities in an integrated manner. Children’s motor competence, including their capacity to execute fundamental motor skills as well as their perceived motor competence, therefore, impacts and is most likely reciprocally impacted by healthy risk-taking. Possessing high perceived or actual motor competence allows children to engage in different interactions with risky environments and tasks compared with children who possess lower perceived or actual motor competence because perception and action are intertwined processes occurring between the individual, the task, and the environment [30,31].

Despite its well-documented importance and the willingness of children to engage in risky play [5,6,32,33], there are indications that this type of play is increasingly restricted [34-37]. ECEC institutions restrict risky play because they fear being sued in case of child injury and they receive pressure from the insurance industry to avoid any injuries [32,35,36,38,39]. Moreover, parents restrict children from playing outdoors because they are concerned about traffic accidents [39-43], that children could be injured or kidnapped, or because they fear rainy or cold weather [39]. When children are prohibited from risky play through overprotection, there may be negative effects on their development.

The downward trend in children’s opportunities for risk-taking in play [44,45] may also have wider negative consequences. First, an increased focus on safety has resulted not only in greater restrictions on children’s movement but also less challenging and engaging play environments [35-37,46-50]. Second, there is concern that lack of play opportunities may result in negative long-term consequences such as reduced well-being and excessive or unhealthy risk-taking (eg, through risky substance use or sexual behavior).

As evidence accumulates concerning the benefits of risky play, both researchers and policy makers have begun to recognize the limitations of our current knowledge. Most existing data are based on descriptive studies and fail to offer rigorous data to address potential causal relationships between risky play and benefits to children’s health, development, and learning. The field hypothesizes that engagement in risky play in childhood may reduce the likelihood of subsequent anxiety, faulty decision-making, and excessive risk-taking later in life. However, those hypotheses are generally based on data from small samples and frequently lack the rigor of longitudinal designs, randomization, and control groups.

One reason for the sparsity of rigorous data is that the hypotheses are difficult to test empirically. There are obvious ethical issues with conducting studies designed to allow or encourage children to take physical risks with the potential of injury. There are also ethical barriers to restricting children from playing freely or to randomly assigning some children to play freely and others not. Thus, assessing the long-term effects of play choices is problematic. Creative methodologies such as Kretch and Adolph’s [51] and Adolph and Kretch’s [52] experiments with the visual cliff (ie, allowing children to cross narrow bridges without the real possibility of falling) or Morgan et al’s [53] laboratory-based activity room to assess behavior should be further developed, perhaps leveraging virtual reality (VR) technology [54,55] as a promising methodology. However, transferability of results from laboratory or other controlled environments to real-life contexts poses a potentially inappropriate leap [56]. There is a need to continue to develop VR, augmented reality, and other technologies to simulate real-life settings and strengthen ecological validity of research methods. Using VR or augmented reality enables researchers to test situations that would be too dangerous to investigate in real life. Adding measurements of bodily movement and eye-tracking equipment, mostly absent in the field, would contribute detailed data on children’s reactions to stimuli in their environments. Understanding connections between risk experiences and risk management is central to understanding how risky play affects overall health and development.

In the Virtual Risk Management—Exploring Effects of Childhood Risk Experiences through Innovative Methods (ViRMa) project, risk is defined as future uncertainties that can entail negative or positive outcomes [44]. Further, risk is seen as both objective and subjective [57,58]. *Objective *risk refers to an estimation of the probability of an adverse event and of the expected harm or loss. The ways in which objective factors are experienced as risky depend on the individuals and are referred to as *subjective risk*. Both personality and temperament traits as well as cognition [59-62] and previous experience [57,63] affect an individual’s subjective risk experiences and decisions. Risk decisions can be interpreted as representing a balance between an individual’s propensity to take risks, the potential rewards of risk-taking, the perceived *danger* in the situation, and *previous experience *with injuries or losses [57].

In the ViRMa project, we aim to explore risks that are relevant to children’s everyday life and that they can at least partly control themselves. These are risks where children’s dynamic choices and actions matter. To capture this and to develop scientific measures, we have developed a conceptual framework (in process to be published in a theoretical paper) to describe the dynamic process through which children manage risks, a framework that captures how the child acts in an environment with potential for risk and where their risk willingness (ie, the emotional drive to seek and take risk, overlapping conceptually with sensation seeking and risk seeking), risk assessments (ie, the cognitive process of evaluating risk throughout a risk situation), and risk handling (the behavioral or motor process of managing a risk) can be observed through their actions and behaviors. Overall, children’s dynamic risk management therefore represents the outcome of a defined task or the observable outcome of whether they fail or succeed in the risk situation.

### Study Objectives

The aim of the overall ViRMa project is to develop, test, and validate ethically appropriate, technologically innovative, and easy-to-use methods to explore the relationship between children’s risk experiences through play and daily activities and their risk management skills. We introduce methods that to our knowledge did not exist before ViRMa. Thus, the project focuses especially on method development and exploration concerning whether the methods are useful and valid to measure the relationships of interest.

This paper describes the protocol of the third stage in the ViRMa project. Stages 1 and 2 had the following aims: (1) develop VR, eye-tracking, and motion-capturing tools and software, plus psychometric scales and additional data collection methods to provide insight into how children assess and handle risk situations using ethical study designs; (2) conduct a small-scale pilot study to test the feasibility of the research methods, particularly the developed VR scenarios, the eye-tracking technology, and the motion capture system.

In the presently reported final stage, the tools and methods developed in stages 1 and 2 will be used for large-scale data collection. The aim of stage 3 is twofold: (1) establish measures for children’s risk management, suggestively split into risk willingness, risk assessment, and risk handling through the VR, eye-tracking, and motion-capturing data, as well as validating the questionnaires to a Norwegian context, and (2) explore how children’s past risk experiences through play and daily activities (collected through parent questionnaires) are associated with their risk management.

In this trial, we are measuring associations between behaviors in VR with demographics as a first step to validate the methods and explore relationships between children’s risk experiences, their motor skills, and their risk management skills. If we succeed with the stage 3 ViRMa project, the long-term goal for the project group is to further develop a large-scale project with children and parents from different countries and cultural contexts, preferably with longitudinal data collection. This would enable both comparative analysis across cultures and longitudinal data on how children’s motor skills and risk management skills develop over time in accordance with their opportunities for risk experiences.

## Methods

### Study Design

This study is a quantitative cross-sectional study of children’s risk management in VR scenarios, measuring their risk willingness, risk assessment skills, and risk handling skills as well as their motor skills. Cross-sectional data on children’s self-perceived motor skills and sensation seeking personality will also be collected. In addition, the study has a retrospective component that gathers extensive data from parents’ questionnaires concerning the children’s background experiences: parenting styles, parental risk tolerance, and children’s practical risk experience from childhood. Analyses will explore associations between cross-sectional data and retrospective data.

### Participants and Eligibility Criteria

We aim to recruit 500 children (50% girls and 50% boys) in second to fourth grade (7- to 10-year-old). This age group was chosen because we want to not only explore risk management skills among children as early in child development as possible but also consider practical matters such as the appropriate age to use current generations of VR goggles given their large size, weight, and functionality. VR technology develops rapidly, and studies involving even younger children would be an aim in the future. Further, children aged 7 to 10 years are likely familiar with navigating traffic and nature environments, while also remaining interested in exploring a playground, which reflects the tasks in the VR scenarios in this study. This is also an age group when children are more likely to behave independently and outside adult supervision, creating greater injury risk.

We aim to recruit children from both urban and rural primary schools in Norway because children in rural areas usually spend more time playing and being active in nature environments than children living in urban areas. The pilot study demonstrated that VR is a popular data collection method among children. To avoid excluding any children from a particular setting, we will select 4 primary schools from different parts of Norway and invite all children in the relevant age range at each school to participate. Inclusion criteria are as follows:

- Between the ages of 7 and 10 years (grades 2, 3, and 4 in Norwegian schools)
- Informed consent obtained from parents to participate
- Assent obtained from the child to participate.

We will contact selected schools through the principal and ask if they are willing to participate. If they agree, parents will then be contacted through the school’s web-based information system, with information about the project and an electronic consent form provided. Children whose parents provide consent will then be placed on a participant list and names will be replaced with a random code. Children will also be asked for assent to participate, and if they decline, their name and code will be deleted.

Parents (both mothers and fathers) of children will be invited to complete the web-based survey.

### Data Collection and Measures

#### VR

VR technology encompasses computer technology enabled by, in this case, head-mounted displays (goggles) in which one experiences and physically moves around in scenarios that closely represent real life. Participants will be fully immersed in the virtual environment, providing visual and auditory stimuli to create illusive feelings of physically existing in that environment [64]. VR technology is an innovative and successful strategy for training and research on health and safety behavior among both adults [64,65] and children, with a large body of child research focused on street-crossing [54,55,66,67] and bicycling tasks [68,69]. In this study, we use VR technology (HTC Vive Pro Eye VR headset), with 4 SteamVR 2.0 Base Stations defining a VR area of 6 × 7 m for free movement, to test how children manage different kinds of risk situations.

On the basis of research into common causes of injury among children [1-3], we developed three categories of VR risk scenarios: (1) street crossing, (2) crossing a river, and (3) Balancing on high playground equipment. All scenarios include various tasks with increasing levels of difficulty and risk.

The VR scenarios were developed through collaboration between the research group and a VR design company (Nordic Neurotech AS) from September 2021 to March 2022. The aim was to create fully immersive scenarios in a virtual world where children performed risky tasks and judged the experience to be as similar to reality as possible. The veracity of the VR scenarios is strengthened through realistic sounds integrated in the VR goggle headphones (eg, city sounds, car sounds, birdsongs, and sounds of running water). In all VR scenarios, children receive the same prerecorded messages before each task, telling them what to do (eg, “Cross the river without falling down”).

The VR software provides data output in the form of a text file (.txt), with one line for each recorded hertz. Within each line, the position of the headset and the 5 Vive trackers are provided using the x, y, and z coordinates. Data on the headset and tracker’s rotation are also available in the data set. Similarly, the position of every simulated moving object (cars and bicycles) is represented with coordinates. Quantitative data from this text file are obtained through MATLAB analysis.

##### Street Crossing

**Figure 1:**
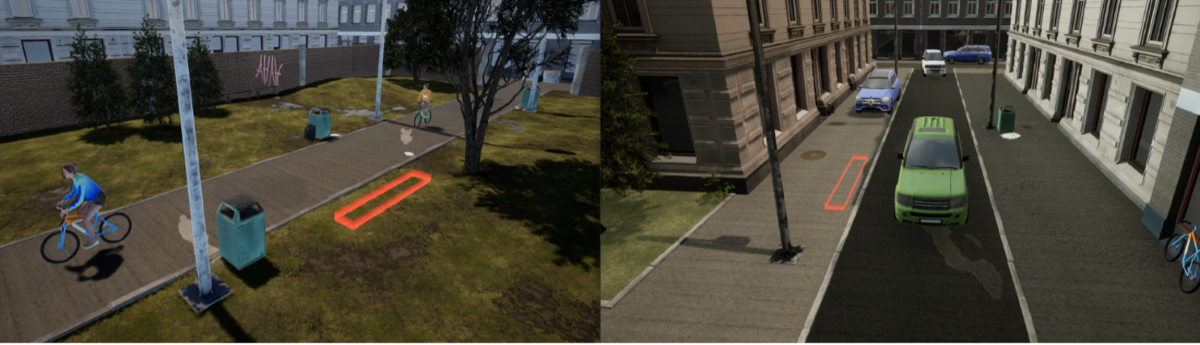
Screenshots of the virtual bicycle pathway environment and the virtual street environment. The red square represents the point where children are asked to move to.

The street crossing scenario consists of 2 urban traffic environments. In both environments, children are exposed to 3 different tasks with varying levels of risk, with risk level varied by manipulating harm severity and probability of being hit [70,71]. The lowest risk scenario involves bicycles and the highest involves both cars and bicycles. Probability risk is adjusted through traffic density and speed and by including traffic from 1 direction versus 2 directions. The first environment represents a bicycle pathway and includes buildings, trees, and grass surfaces (Figure 1, left side). The second environment replicates a busy urban street with buildings, sidewalks, and parked cars (Figure 1, right side); it includes a cars-only task as well as tasks with both cars and bicycles. In all cases, the child’s goal is to cross the path or street without getting struck by a car or bicycle. All street-crossing scenarios incorporate realistic sounds from the urban environment (the sound of cars, bikes, people, etc).

If the child is hit by a bike or car, a new trial on that same task is provided. If the child is hit a second time, the VR continues to the next task. The time to complete tasks in the street-crossing scenario will vary across children because they will be free to take the time they need, both to assess the situation before acting and to perform the crossing. Pilot research found that children spent on average 3 minutes to complete the street-crossing tasks (Figure 1, left and right side).

Quantitative data points that will be obtained from the street-crossing tasks include measures related to the child’s risk assessment (eg, time used to assess the environment before crossing the street and fixation on vehicles before crossing the street), risk willingness (eg, distance to vehicles when starting to cross the street), risk handling (eg, speed of crossing, running, acceleration, and deceleration), and risk management outcomes (eg, number of times the child was hit or almost hit by vehicles).

##### River Crossing

The river-crossing scenario presents a rural environment with a flowing river. Children complete 7 tasks, each with the goal of crossing the river without falling into the water. Five of the tasks involve balancing on planks to cross the river, whereas in the other 2 tasks children must step or jump between elevated stones to cross. Similar to the street-crossing scenario, the 7 river-crossing tasks vary in risk, based on harm severity and probability of failure [70,71]. Harm severity risk levels are manipulated by the speed and depth of the river and the presence of rocks in the water. Probability risk is adjusted by the width (between 8 and 38 cm) of the balance planks and the placement of stepping stones (ie, how close or far they are from each other). In all river-crossing scenarios, children hear sounds from a rural nature environment (eg, the sound of running water, wind, and birds).

**Figure 2:**
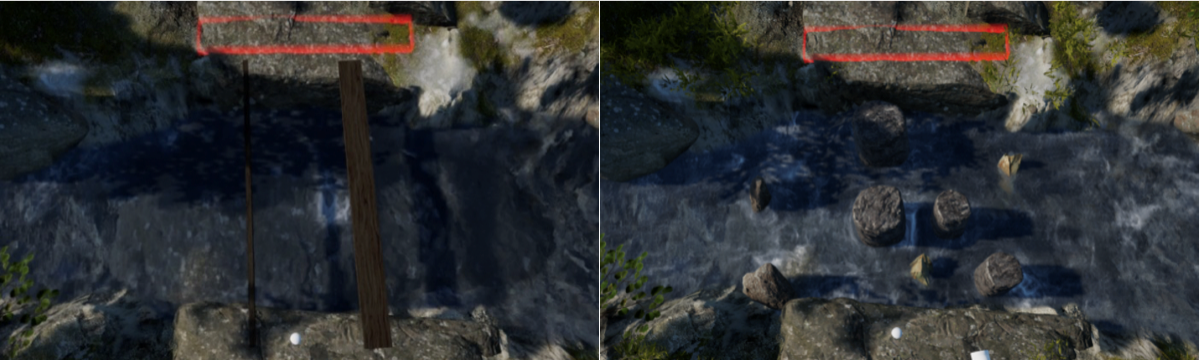
Screenshots of the river crossing scenario, one of the plank tasks and one of the stepping stones task.

If the child falls into the water, a new trial on that same task is provided. If the child falls a second time, the VR continues to the next task. The time required to complete the river-crossing scenario tasks will vary between children because they are free to take the time they need, both to assess the situation before acting and to perform the crossing. Pilot research found that children spent on average 3 minutes to complete the river-crossing tasks (Figure 2).

Quantitative data points that will be obtained in the river-crossing tasks include measures related to the child’s risk assessment (eg, time used to assess the environment before crossing the river), risk willingness (eg, opting out of the task, jumping between rocks, and choosing the narrow plank), risk handling (eg, speed of crossing, keeping both feet on the ground while balancing, acceleration, and deceleration), and risk management outcomes (eg, number of times the child falls into the river).

##### Playground

**Figure 3:**
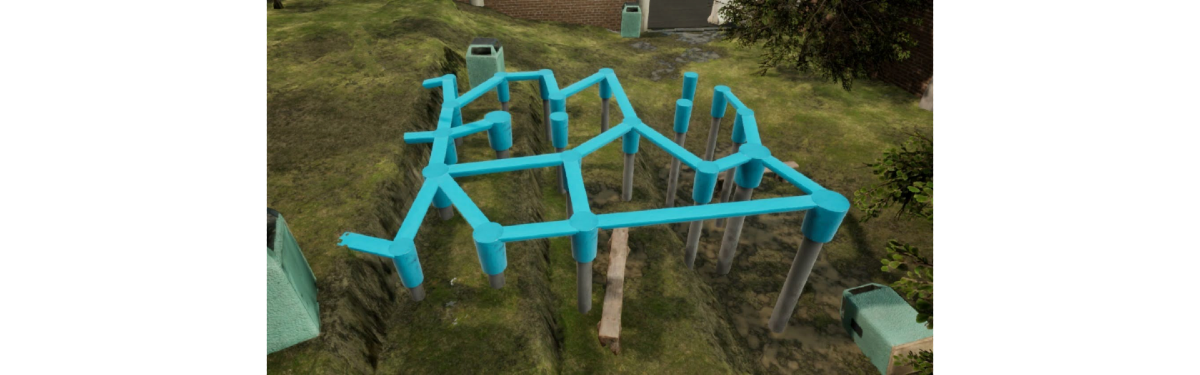
Screen shot of the playground scenario.

The playground scenario presents a balancing play structure in an urban playground environment. Unlike the first 2 scenarios, where children perceive a fixed and objective task to cross the street or river, in the playground scenario, children are free to move and play as they like, without fixed objectives or goals. The virtual balancing structure comprises a complex pattern of balance beams of varying widths, vertical pillars of varying diameters, and 4 different height zones above the ground surface. Similar to the first 2 scenarios, variation in harm severity and probability of failure occur within the playground scenario. Probability risk varies by the difficulty of the play areas (ie, a child could choose to balance on the narrowest beams or jump out to one of the pillars), whereas harm severity varies by the height zones children choose to explore. In the study, children are provided 3 minutes to explore the playground before the scenario automatically ends. If a child falls off the playground equipment, he or she will enter the starting point of the playground task and have the opportunity to further play and explore during the remaining time in the 3-minute period. The sounds of an urban environment (eg, cars, bikes, or people) are emitted through the headphones (Figure 3).

Quantitative data points that will be obtained during the playground task include measures related to the child’s risk assessment (eg, time used before starting to balance on a beam), risk willingness (eg, stepping out on pillars, jumping over gaps, and time spent in high-risk zones), risk handling (eg, speed of balancing, keeping both feet on the ground while balancing, acceleration, or deceleration), and risk management outcomes (eg, number of times the child falls off the playground equipment).

#### Eye Tracking

Eye-tracking technology is used extensively in research to explore topics such as how humans learn [72] and how humans use their gaze when performing safety practices [73], as well as for medical training (eg, in laparoscopic surgery or when detecting anatomical injuries) [74]. The technology is also used to measure how children’s eye movements are related to their performance, risk, and safety behavior when cycling [69]. In street-crossing VR tasks, eye-tracking data from one study demonstrated that children’s ability to focus on factors most salient for a safe crossing is compromised when the environment is more visually loaded, possibly causing them to miss critical information [75]. However, knowledge about the relationships between children’s eye movements and their risk management is limited.

**Figure 4:**
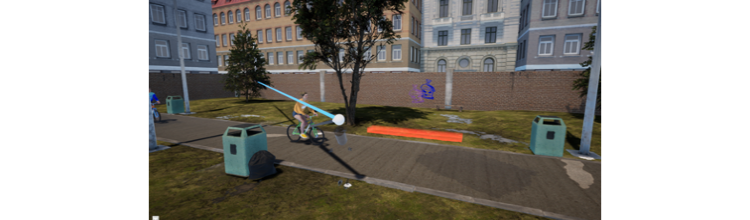
Screen shot from the bicycle path scenario, showing eye-tracking as a blue line.

In this study, we will use eye-tracking technology (Tobii Pro) integrated in the VR goggles to explore how children use their gaze and eye movements to identify risk factors that guide their navigation, bodily movement, and risk decisions in the VR scenario environments. Eye-tracking data will be logged in 90 Hz and provide data on the eye-tracking target (x, y, and z coordinates), including what object the child looks at. Because this information is provided continuously with a refresh rate of 90 Hz, it can be transformed into variables describing the length of time children look at various objects or parts of the environment, direction, and shifts of the child’s focus and information about how children follow moving objects such as vehicles with their eyes (Figure 4).

The eye-tracking data from all VR tasks will be processed in MATLAB to generate quantitative measures related to the child’s risk assessment (eg, fixation on vehicles before crossing the street, looking at oncoming or departing bicycles and cars before crossing, looking both ways before crossing the street, looking at planks and balance beams before and while crossing, looking at water and rocks, and looking at the ground below the playground equipment) and risk handling (eg, looking at oncoming bicycles and cars while crossing).

#### Motion Capturing and Analysis

Part of the innovative approach of this project is assessing children’s motor competence when they navigate and explore VR scenarios. We will accomplish this through whole-body motion capture, which offers a comprehensive understanding of the individual’s qualitative and quantitative signatures of movement. This methodology represents a novel approach toward the assessment and understanding of motor competence in children, extending current and predominant approaches that focus on tasks with down-scaled complexity, detailed instructions, and highly specific assessment criteria. Existing approaches have been critiqued for their lack of developmental and ecological validity [76].

The Xsens MTw Awinda system of motion-capturing technology is an innovative and user-friendly method to track human motion in real time. The portable system provides accurate time-synchronized data with accurate measures of whole-body movements. It has been used widely in research to capture human motion in sport, physical activity, and rehabilitation [77-80]. In this project, MTw Awinda is used to track and capture children’s motion and bodily movements when handling risk in VR scenarios.

The Xsens MTw Awinda system consists of 17 wireless inertial measurement units, each of which consists of a gyroscope, an accelerometer, and a magnetometer. MTw Awinda samples data at 60 Hz through a proprietary radio protocol at 2.4 GHz, which provides accurate time synchronization across the wireless network within 10 µs. Physically, the inertial measurement units are 47 × 30 × 13 mm, weigh 16 g, and are attached to the children with Velcro straps on the following anatomical locations: forehead, sternum, palmar side of the hand (right or left), lateral side of the upper arm (right or left), wrist (right or left), lateral side of the lower leg (right or left), lateral thigh (right or left), foot (right or left), upper part of the scapula (right or left), and lower back (L5, height of the iliac spine). The system provides 3D kinematical data (position, velocity, acceleration, and orientation) on the movement of 23 different segments (eg, head, upper hand, or foot) as well as angular movements of 22 different joints. The dynamic accuracy is 0.75° root-mean-square (RMS) and 1.5° RMS for roll or pitch and heading, respectively. In the ViRMa project, children will wear the Xsens MTw Awinda sensors while completing all VR tasks.

The raw Xsens data will be exported and further processed in MATLAB R2022a (MathWorks Inc) by algorithms developed by the research group. After inspection of the frequency spectrum content of the raw data with the periodogram method, low-pass (zero-phase) Butterworth filtering procedures will be applied to remove unwarranted noise. To obtain numeric measures related to children’s motor competence and risk handling strategies (ie, bodily movement strategies), 2 main approaches to movement analysis will be applied. First, analysis of children’s overall locomotory strategies, defined as the movement (transport) of the entire body as children navigate the VR scenarios, will be conducted by examining the 3D kinematic variability of the pelvis segment (ie, the location of the body center of mass). This analysis generates variables on various motoric risk handling strategies, such as velocity and acceleration profiles (ie, speeding up or slowing down), angle of projection when moving across the road, and degree or pattern of postural movements to maintain balance and stability. In the second approach, the kinematic variability of the major joints from the whole body (ie, neck, shoulders, elbow, hip, knee, and ankle) will be analyzed. Here, numeric measures closely associated with motor competence, such as degree of repetitiveness and variation in joint movements (entropy), association between upper body versus lower body movements, and interjoint coordination patterns, will be examined.

### Child Questionnaires

#### Children’s Sensation Seeking

Sensation-seeking personality, defined as an innate willingness to take risks and the extent to which children seek risk in their everyday life, will be assessed using the Sensation Seeking Scale for Young Children (SSSYC) [81]. A 27-item instrument, the SSSYC was developed to assess sensation seeking among children aged 7-12 years. Each item presents 2 statements of preferences that child respondents choose between. As an example, children are presented the following options: (1) *I am the sort of person who would like to sled fast down a steep hill* compared with (2) *sledding fast down a steep hill sounds scary to me*. The SSSYC includes three subscales, each with strong evidence for internal reliability: (1) thrill seeking (α=.85), (2) behavioral inhibition (α=.83), and (3) behavioral intensity (α=.83) [81]. Selecting the statement indicating higher levels of the measured subscale earns 1 point on the overall subscale score. In the above example, selecting the first option earns 1 point on the thrill-seeking scale, whereas choosing statement 2 earns 0 points.

For this study, the SSSYC was first translated from English to Norwegian by author OJS. Then all primary researchers in the project group discussed the translation to revise unclear wordings and adapt words and phrases to fit the Norwegian context. Next, the Norwegian version of the scale was back-translated to English by a professional translator, and then the 3 versions (original English, translated Norwegian, and back-translated English) were discussed among the project group to ensure that the original meaning of the statements was reflected in addition to being relevant and understandable for Norwegian children. In the ViRMa project, we will administer the SSSYC orally, with a researcher recording the child’s responses on the web.

#### Children’s Perceived Motor Competence

The Pictorial Scale of Perceived Movement Skill Competence [82] will assess children’s perception of their own motor competence. In this 18-item measure, children view 2 drawings of various movements or activities (eg, climbing up a rope or riding a kick scooter) in each item. In one drawing, the child manages the task well, and in the other, the child does not manage the task well. Children are asked which picture most resembles themselves and their skills and also to indicate their perceived competence in that skill as (1) really good, (2) pretty good, (3) sort of good, or (4) not too good. The scale has separate boys’ and girls’ versions, with three reliable subscales resulting (1) Active Play (α=.78), (2) Object Control—Hand Skills (α=.76), and (3) Fundamental Movement Skills with Leg Action (α=.84) [82].

For this study, the Pictorial Scale of Perceived Movement Skill Competence was translated from English to Norwegian by the author HL. Then, all primary researchers in the project group discussed the translation to revise unclear wordings and adapt words and phrases to fit the Norwegian context. Because this scale is primarily based on drawings and not words, it was not back-translated. In the ViRMa project, the Pictorial Scale of Perceived Movement Skill Competence will be conducted orally, with the drawings shown to the child on a tablet.

### Parents Questionnaires

#### Parents’ Tolerance of Risk in Children’s Play

The Tolerance of Risk in Play Scale (TRiPS) [83] will be used to gather data on parents’ tolerances of risk during their children’s play. The TRiPS is a 31-item scale focusing on parents’ willingness to let children take risks in play. Parents select “yes” or “no” for questions such as “Would you let your child jump down from a height of 3-4 meters (10-13 feet),” or “Would you allow your child to play on equipment if you thought there was the potential s/he may break a bone?” The original English version of the TRiPS was validated using Rasch analysis and found that all items had mean square values within an acceptable range for internal (construct) validity and that internal reliability statistics were excellent (showed a high person reliability index of 0.87) [83].

For this study, the TRiPS was first translated from English to Norwegian by the author EBHS. Then, all researchers in the project group discussed the translation to revise unclear wordings and adapt words and phrases to fit the Norwegian context. Next, the Norwegian version of the scale was back-translated to English by a professional translator, and the 3 versions (original English, translated Norwegian, and back-translated English) were discussed in the project group to ensure that the original meaning of the statements was retained in addition to being relevant and understandable for Norwegian children. In ViRMa, the TRiPS will be administered to all participating children’s parents, both mothers and fathers, via a web-based platform using the survey tool SurveyXact [84].

#### Parenting Style

The Challenging Parenting Behavior Questionnaire (CPBQ) [85] will be used to assess the extent to which parents provide physical and verbal messages that encourage children to push their physical and mental limits. Parents are presented with 33 statements, such as “I play boisterously with my child” and “I encourage my child to do exciting things, such as jumping off high objects or climbing higher than he/she dares,” which are answered on a scale from 1 (not applicable) to 5 (completely applicable). The instrument includes six subscales: (1) Teasing, (2) Rough-and-tumble play, (3) Encouragement of risk-taking, (4) Social daring, (5) Competition, and (6) Modeling. Internal reliability of the full scale ranges from α=.90 to α=.92 in samples of Dutch and Australian mothers and fathers, and the subscale reliabilities range between α=.63 and α=.84 in the same samples [85].

The original CPBQ was written in Dutch and translated to English using standard rigorous translation methods. For this study, the CPBQ was first translated from English to Norwegian by the author RK. Then, all primary researchers in the project group discussed the translation to revise unclear wordings and adapt words and phrases to fit the Norwegian context. Next, the Norwegian version of the scale was back-translated to English by a professional translator. The back translation to English was then discussed with Mirjana Majdandžić, who developed the original Dutch version, to ensure that the Norwegian version covered the meaning content of the original scale while still being relevant and understandable for Norwegian children. The project team also reviewed the back translation version in comparison with the original English version. In ViRMa, the CPBQ will be administered to all participating children’s parents, both mothers and fathers, via a web-based platform using the survey tool SurveyXact [84].

#### Background and Demographic Information

To gather background and demographic information, children’s parents, both mothers and fathers, will respond to items about children’s age, ethnicity, past injury history, living conditions (urban vs rural), access and use of nature, modes of traveling to school, participation in extracurricular physical activity (eg, sports), ECEC experience, family socioeconomic background, and other relevant information. The survey will be administered along with the TRiPS and CPBQ via a web-based platform using the survey tool SurveyXact [84].

During the study, children will respond to questions to capture their experiences and feelings while participating in the VR scenarios. For example, they will be asked, “Did you feel it was for real?” and “Have you ever tried VR before?”

### Data Collection Procedure

We will collect data in each school over the course of 2 to 3 weeks. We will install the VR equipment in a dedicated large room at each school, with enough space for a 7 × 6 m test area, with 4 SteamVR 2.0 Base Stations placed in each corner and room for children’s free movement within the space. The test area, with all 4 base stations as well as the VR goggles, will be calibrated with the normal calibrating procedures in SteamVR 2.0. We will identify participating children individually in their classrooms and escort them to the test area. Sensors (17 Xsens sensors) and trackers (5 Vive trackers, version 3) will be placed on the child’s body using Velcro bands. Next, we will introduce the child to the VR goggles and ensure that goggles are placed correctly on the head. VR goggles placement and eye-tracking measure sensitivity will then be calibrated through procedures in SteamVR 2.0. The child will enter a warm-up scenario in the virtual environment where they can explore a city park and familiarize themselves with being within, and moving around, the virtual world. During the warm-up, we will calibrate the Vive trackers to enable children to see their feet and also calibrate the Xsens sensors to ensure that motion-capturing measurements are correct. The last part of the warm-up scenario instructs the child about safety routines, as follows:

You will now be taken to three different scenarios where you should perform different tasks. The first scenario shows several traffic situations, including cars and bikes, where your task is to cross the street without being hit. The second scenario is a river in the forest where your task is to cross the river without falling into the water. Some tasks include balancing on a beam and some include stepping on stones to cross the river. The last scenario is an urban playground where you are allowed to do whatever you want and to play and explore the playground equipment as you like, for three minutes. I will be here walking next to you to make sure you don’t trip on the cable (attached to the goggles). If you ever want to quit the trial or skip to the next task, you can just let me know and I will help you.

When the child indicates that they are ready to start the test, we will initiate the surround-sound headset for further verbal instructions, and the child will enter the first and simplest street-crossing scenario task (ie, bicycle pathway). The verbal instructions built into the headset when entering each task are simple.

- Street crossing: “Cross the road without getting run over.”
- River crossing: “Cross the river without falling down.”
- Playground: “Here you can move around on the playground, explore if you want, and try not to fall down.”

Risk levels increase with each scenario as described previously. Children will complete all tasks in each scenario in the following order: street crossing, river crossing, and playground. The pilot (stage 2 of the overall project) found that completing the warm-up or calibration and all tasks in all scenarios took approximately 15 minutes for each child.

After the child has completed all VR scenarios, we will remove test equipment from their body and ask them to join a researcher for a short interview. During the interview, which will last approximately 15 minutes, the researcher will use a tablet to complete the child scales and background questions orally with the child. Parents will receive the survey via email with a web-based link that includes a code that matches their child’s code to enable matching the dyad’s data.

Three researchers will conduct the data collection procedures in each school. The group of data collectors consists of 5 researchers (EBHS, OJS, HL, RK, and LS) who are well trained, have developed all the primary and secondary outcome measures in this study, and designed and prepared (including piloting the implementation of all test procedures) the study protocol.

### Ethics Approval

The Norwegian Social Science Data Services approved the ViRMa project (project 324155). We treat all data and information related to the project in accordance with the ethical guidelines of Norwegian Social Science Data Services. Child-friendly methods of seeking informed assent from children will be used, and informed consent will be obtained from the children’s parents or guardians. We will take careful measures to avoid placing research participants at risk by being sensitive to the specific challenges that arise in connection with involving children in research, as well as securing their right to withdraw from the research at any point. In the ViRMa project, we emphasize ethical responsibility in the presentation of all research results involving research participants in the project.

One possible undesirable effect of carrying out the data collection with VR scenarios is that children may become frightened of the simulated situations they encounter. A second risk is that children are overconfident, overestimate their risk management skills, and experience virtual falls in the VR scenarios that they find traumatic. Our pilot research suggested that these outcomes are both unlikely, but to address them, we will debrief all participating children, discuss their experiences, and remind them that their performance in VR tasks does not reflect reality.

### Analysis

The measures obtained from the VR, eye-tracking, and Xsens data include quantitative variables related to the child’s risk assessment, risk willingness, risk handling, and risk management. This study’s explorative and groundbreaking nature implies that an essential part of the analysis is to develop and validate new measures based on VR, eye tracking, and motion capturing. Consequently, the plan for data analysis is relatively general. The study therefore sets a conservative significance level (*P*<.01) to reduce the risk of type 1 error.

Cluster analyses will be applied to group children’s movement profiles based on the Xsens data divided into potential distinct groups by comparison of hierarchical agglomerative clustering methods: the Ward method and the between-groups average linkage method. For both methods, the squared Euclidean distance will be used as the measure of proximity. The final number of clusters will be determined by examining the agglomeration schedules and dendrograms generated for both techniques. Visual inspection of each cluster’s membership will be conducted to assess the utility of each potential cluster solution. Once the most statistically robust and theoretical relevant cluster solution is identified, a K-means iterative partitioning method will be applied to fine-tune the clusters [86,87].

Global measures on the child’s risk assessment, risk willingness, risk handling, and management will be established using exploratory factor analysis [88] drawing on data from VR, eye tracking, and motion capturing from the VR street crossing, river crossing, and playground tasks. RMS error of approximation (RMSEA), standardized root-mean-square residuals (SRMR), comparative fit index (CFI), and Tucker-Lewis index (TLI) will be used to evaluate and refine the established measurement models. Acceptable model fit is defined as RMSEA <0.1, SRMR <0.1, CFI >0.9, and TLI >0.9 [89]. Chi-square and chi-square difference tests will be conducted between the fitted and saturated models. However, no absolute thresholds for chi-square are set, following the sensitivity to sample sizes and the possibility that this measure may yield less precise estimates of the parameters [88,90]. Factor loadings will be investigated with the use of *R*² estimates (item >0.25) and standardized factor loadings (item >0.40) [88]. To validate the psychometric scales in the Norwegian context, factor analysis will be used. The scales with predefined subscales (SSSYC, Perceived Movement Skill, and CPBQ) will be validated using confirmatory factor analysis, and the TRiPS will be evaluated using exploratory factor analysis [88]. Acceptable model fit is defined as RMSEA <0.1, SRMR <0.1, CFI >0.9, and TLI >0.9. Factor loadings will be investigated with the use of *R*² estimates (item >0.25) and standardized factor loadings (item >0.40) [88].

Because the participating children are nested within schools, generalized structural equation modeling and multilevel regression analysis will be used in the primary outcome analysis to explore how children’s past risk experiences through play and daily activities are associated with their risk management. Children’s past risk experiences through play and daily activities will be measured primarily through the CPBQ and TRiPS, whereas measures related to the child’s risk management will be obtained from global measures related to risk management from the VR simulation. The child’s age, sex, and other relevant background and demographic information, as well as their sensation-seeking personality (measured through the SSSYC), will be included in the models.

## Results

Funding to conduct the ViRMa project was confirmed in June 2021. From August 2021 to February 2022, the project group developed the VR scenarios, including eye-tracking technology and motion capturing, translated the scales, and developed the questionnaires. We will conduct the data collection from January 2023 to May 2023.

Four schools were recruited as data collection sites, with a total of approximately 660 students in second to fourth grade (7-10 years old). We aim to enroll 500 children and their parents for participation. The recruitment of children and parents for this study started in December 2022, and as of April 2023, a total of 433 parents consented for their children to participate.

## Discussion

### Principal Findings

This study will increase our understanding of child development. An essential goal of many educational, developmental, and pediatric health research studies is to understand how children’s characteristics, upbringing, and previous experiences influence their learning and ability to handle challenges. This study focuses on children’s abilities to manage risky scenarios and ties this to previous experiences. Using cutting-edge technology and previously developed measures to describe aspects of the child’s past experiences, this project addresses crucial topics related to children’s health and development. Such knowledge may guide pedagogical questions and development of educational and health-related interventions and reveal essential areas for focus in future studies.

One novelty of this study is related to technological and methodological advancements that may lead to new knowledge and discovery. The development and use of VR tools to explore how children handle real-life scenarios opens numerous avenues of research in different scientific fields. The integration of VR, eye-tracking, and motion-capturing technology has not been presented in published research to date and addresses many present scientific challenges. Moreover, the research represents methodological development in how VR technology may be paired with various measures in real time. Through life-like VR scenarios, it is possible to investigate children’s risk-taking in a controlled and safe environment, which may lead to substantial theoretical and empirical progress. A key ambition of the study is to develop and use technology and methods in a way that enables large-scale, replicable, international studies in other cultural contexts. Thus, the tools and technology developed must be standardized and easy to use.

From the perspective of contributions to developmental science, the project can lead to further scientific understanding of children’s motor competence and how it can be assessed. The combination of whole-body motion capture and VR embedded with tasks in which children can move around to explore and complete tasks represents a completely new research approach. With these features, children’s motor competence can be examined through the lens of movement behaviors emerging when individual, task, and environmental components interact. This strategy circumvents shortcomings identified within existing motor competence assessments and has broad implications for further work in designing and evaluating interventions and programs, as well as for research into underlying developmental processes and the role of influencing factors in the adaptive change within individuals as they work toward motor competence throughout childhood and adolescence.

The ViRMa project results will also benefit societal goals related to health promotion, education, parenting, and city and regional planning. A cross-cutting trend today is related to risk aversion resulting from the fear of injuries to children. Compelling theoretical perspectives suggest that reducing children’s possibilities to engage in risky situations may be counterproductive in terms of preventing injuries because children’s opportunities to learn to estimate and manage risk are diminished. If this study establishes empirical support for this hypothesis, it may impact how risk is addressed in crucial societal institutions like the family, early childhood education, and schools. Specifically, the ViRMa project aims to develop knowledge that may lead to healthier lives and fewer injuries through new knowledge about how children best learn to judge and manage risky situations in playful contexts. Through the acquisition of knowledge about children’s risk management in play, this study may benefit societal challenges related to children’s health and development and reduce economic burden to society stemming from the cost of unintentional injury treatment among children and young people [91].

### Strengths and Limitations

The use of VR tools along with integrated eye-tracking and motion-capture technology to explore in real time how children handle real-life scenarios in a safe context opens numerous possibilities to address scientific challenges and answer critical gaps in both the scientific literature and application of science for societal needs. However, our ambition to develop and use scientific methods that can be used in various contexts with portable data collection equipment comes with limitations. The test setup might be incrementally more accurate and valid in a controlled laboratory setting where the equipment could be mounted permanently, and the surrounding environment is ideal for the Vive equipment. In a standardized laboratory setting, the simulated planks we use might be replicated with actual wooden planks on the floor that are positioned accurately to match the VR simulation using trackers. Such measures would improve the realism of the balancing tasks but were not possible given this study’s mobile and cost-efficient methodology.

The VR tasks we use in this project are newly developed and have not been validated against children’s behavior in real life. Such a study would shed light on a critical question: to what degree does children’s behavior in risky VR tasks replicate their behavior in similar real-life tasks? Previous work studying children’s pedestrian behavior suggests that behavior in VR can replicate real-world behavior [67], and we dedicated extensive work to ensure and validate that the VR tasks were as realistic as possible; thus, we suspect this limitation is minimal.

Although one of our fundamental interests in this study is to explore how past childhood experiences influence children’s risk management, the study design is cross-sectional. Using only retrospective data to assess children’s past experiences with risky play and risk-taking activities could constitute a risk to reliability and validity of the data as recall and response bias could influence responses. This strategy only establishes association, rather than causation, in any statistical analysis. Nevertheless, this study’s solid theoretical foundation and rich methodology ensures the potential to generate casual hypotheses that will advance our understanding of how children develop risk management skills and can be tested in future controlled, randomized, or longitudinal studies.

Other limitations are related to sample recruitment strategies. Participating children are all Norwegian children, and they are enrolled based on their schools’ and parents’ willingness to participate. The sample is not recruited randomly. Norwegian children are believed to engage in more risky play behaviors than children in many other cultural contexts [12,14], which may influence the results. We have chosen recruitment through schools to get a high response rate among the selected age groups and to carry out effective data collection generating an adequate number of participants for sufficient statistical power. Nonetheless, the recruitment strategy has consequences for generalizability of the sample, and the background of the participating children and the lack of randomization in sampling must be considered when generalizing findings to other contexts.

## Abbreviations

CFI: comparative fit index
CPBQ: Challenging Parenting Behavior Questionnaire
ECEC: early childhood education and care
RMSEA: root-mean-square error of approximation
SRMR: standardized root-mean-square residuals
SSSYC: Sensation Seeking Scale for Young Children
TLI: Tucker-Lewis index
TRiPS: Tolerance of Risk in Play Scale
ViRMa: Virtual Risk Management—Exploring Effects of Childhood Risk Experiences through Innovative Methods
VR: virtual reality

## Data Availability

The data sets generated or analyzed from this study are available from the corresponding author on reasonable request.
